# Directed Evolution of *Methanomethylophilus alvus* Pyrrolysyl-tRNA Synthetase Generates a Hyperactive and Highly Selective Variant

**DOI:** 10.3389/fmolb.2022.850613

**Published:** 2022-03-09

**Authors:** Jonathan T. Fischer, Dieter Söll, Jeffery M. Tharp

**Affiliations:** ^1^ Department of Molecular Biophysics and Biochemistry, Yale University, New Haven, CT, United States; ^2^ Department of Chemistry, Yale University, New Haven, CT, United States

**Keywords:** directed evolution, PANCE, PylRS, noncanonical amino acid, tRNA, orthogonal, synthetic biology, pyrrolysyl-tRNA synthetase

## Abstract

Pyrrolysyl-tRNA synthetase (PylRS) is frequently used for site-specific incorporation of noncanonical amino acids (ncAAs) into proteins. Recently, the active site of *Methanomethylophilus alvus* PylRS (*Ma*PylRS) has been rationally engineered to expand its substrate compatibility, enabling the incorporation of difficult ncAAs. However, mutations beyond the active site that enhance the enzymatic properties of *Ma*PylRS have not been reported. We utilized phage-assisted non-continuous evolution (PANCE) to evolve *Ma*PylRS to efficiently incorporate *N* ^ε^-Boc-l-lysine (BocK). Directed evolution yielded several mutations outside of the active site that greatly improve the activity of the enzyme. We combined the most effective mutations to generate a new PylRS variant (PylRS_opt_) that is highly active and selective towards several lysine and phenylalanine derivatives. The mutations in PylRS_opt_ can be used to enhance previously engineered PylRS constructs such as *Ma*PylRS_N166S_, and PylRS_opt_ is compatible in applications requiring dual ncAA incorporation and substantially improves the yield of these target proteins.

## Introduction

The genetic code consists of 61 triplet codons that code for 20 canonical amino acids, as well as three stop codons that function as termination signals to end translation and release the protein from the ribosome. However, exceptions to this rule are apparent, and the plasticity of translation is well-founded. A naturally occurring example is found in several methanogenic species of archaea and bacteria. In these organisms, pyrrolysine (Pyl) is encoded in the active site of methylamine methyltransferase by the amber stop codon UAG (Hao et al., 2002; Srinivasan et al., 2002; Polycarpo et al., 2004). This is accomplished *via* pyrrolysyl-tRNA synthetase (PylRS), a class II aminoacyl-tRNA synthetase that aminoacylates its cognate tRNA, tRNA^Pyl^ (Srinivasan et al., 2002; Polycarpo et al., 2004). This unique tRNA features a CUA anticodon, which enables Pyl-tRNA^Pyl^ to suppress UAG and insert Pyl into the elongating polypeptide through the same translational machinery as canonical tRNAs (Théobald-Dietrich et al., 2004; Zhang et al., 2005; Longstaff et al., 2007).

The PylRS/tRNA^Pyl^ system has been utilized in numerous studies to genetically encode ncAAs at a stop codon (most frequently UAG), altering the target protein’s structure and/or function (Budisa, 2005; Wan et al., 2014; Crnković et al., 2016; Tharp et al., 2018). To that end, PylRS has been developed and implemented to incorporate a wide variety of ncAAs, including *N* ^ε^-substituted lysine (Polycarpo et al., 2006; Neumann et al., 2008; Hancock et al., 2010; Umehara et al., 2012) and, to a lesser extent, *ortho-* and *meta-*substituted phenylalanine (Wang et al., 2011; Ko et al., 2013; Tharp et al., 2021). One of the critical characteristics of the PylRS/tRNA^Pyl^ system is its orthogonality with both bacterial and mammalian host translation machinery (Wan et al., 2014). PylRS/tRNA^Pyl^ can also be used in tandem with mutually orthogonal genetic code expansion tools to incorporate multiple ncAAs into a single protein, such as the *M. jannaschii* TyrRS/tRNA^Tyr^ system. These genetic code expansion tools have been used together to site-specifically incorporate two ncAAs into a single protein in response to two stop codons, most frequently UAG and UAA (Wan et al., 2010; Chatterjee et al., 2013).

*Methanomethylophilus alvus* PylRS (*Ma*PylRS) is a highly active PylRS homolog. Like its previously characterized counterparts, the ncAA specificity of *Ma*PylRS has been modified through active site engineering (Meineke et al., 2018; Willis and Chin, 2018; Beránek et al., 2019; Seki et al., 2020; Tharp et al., 2021). *Ma*PylRS belongs to the class of PylRS proteins that lacks the poorly soluble N-terminal domain. This confers an advantage to *Ma*PylRS, as *Ma*PylRS is far more soluble than full-length PylRS homologs such as *M. mazei* PylRS (Yanagisawa et al., 2008; Seki et al., 2020). The high activity, solubility, and tunable specificity of *Ma*PylRS position it as a powerful genetic code expansion tool.

Improving the activity or altering the substrate specificity of aminoacyl-tRNA synthetases such as PylRS can be approached in two ways: rational engineering or randomized selection (Vargas-Rodriguez et al., 2018; Krahn et al., 2020). Rational engineering typically relies on structural information to guide the process of mutating key residues in the amino acid binding pocket (Wang et al., 2012; Seki et al., 2020). This approach has been used successfully and is straightforward to execute; however, there are likely many targets for mutation that can have a dramatic impact on activity but are easily overlooked during the rational design process. Conversely, randomized selection through library screening or directed evolution is unbiased and can result in powerful mutations that would likely never have been considered for rational engineering (Bryson et al., 2017; Vargas-Rodriguez et al., 2018; Baumann et al., 2019). One such method of randomized selection is phage-assisted non-continuous evolution (PANCE) (Esvelt et al., 2011; Suzuki et al., 2017; Miller et al., 2020). Unlike library-based evolution methods, PANCE does not require structural information to guide selection. PANCE is also simple to implement, as no specialized equipment is necessary. The technique offers tunable stringency and has been shown to generate highly active mutant variants of enzymes such as PylRS. Indeed, PANCE and its continuous evolution counterpart PACE have been successfully used to evolve highly active variants of chPylRS, an enzyme derived from a fusion construct of *M. mazei* and *M. barkeri* PylRS (Bryson et al., 2017; Suzuki et al., 2017).

In this study, we utilized PANCE to evolve *Ma*PylRS with the goal of developing a hyperactive PylRS variant that recognizes a broad spectrum of ncAAs while still discriminating against the canonical amino acids. Sequencing of evolved PylRS variants revealed a polymorphic population of mutations, almost all of which were located outside of the active site. We screened the activity of the mutants towards a variety of Lys- and Phe-ncAAs using *in vivo* fluorescence and chloramphenicol acetyltransferase assays, and we combined the most active mutations to create a new PylRS variant, PylRS_opt_. Our data indicate that PylRS_opt_ excludes canonical amino acids and recognizes a diverse pool of ncAAs, incorporating them with vastly improved activity compared to wild-type *Ma*PylRS. In combination with the *M. jannaschii* TyrRS, the high activity and selectivity of PylRS_opt_ enables robust incorporation of multiple ncAAs into a single protein, which has applications including bioorthogonal click chemistry (Wan et al., 2010; Meineke et al., 2020), peptide cyclization (Neumann et al., 2010; Hayes et al., 2021), and FRET (Wu et al., 2012).

## Materials and Methods

### PANCE

*Ma*PylRS was cloned into M13 phage, replacing gene III. Phages were initially propagated without selection using the permissive host *E. coli* S1059. Cultures were grown in 2xYT media supplemented with the appropriate antibiotics at 37°C until reaching an OD_600_ of 0.4–0.6. Cells were then infected with a viral load of phage ranging from 10^4^–10^6^ pfu/ml, and the culture was grown overnight at 37°C. The following day, a 1 ml aliquot of the overnight culture was centrifuged at 14,000 × g for 1 min, and the phage-containing supernatant was decanted and stored at 4°C. Three independent phage lineages were maintained throughout the PANCE process.

Positive selection was performed using *E. coli* S1030 cells transformed with the accessory plasmid pJT017 (gIII_1xTAG_ *Ma*tRNA^Pyl^_(6)_), as well as mutagenesis plasmid MP4 during rounds of mutagenesis. 30 ml cultures were grown in 2xYT media supplemented with 20 mM glucose and the appropriate antibiotics to an OD_600_ of 0.4–0.6. BocK (5 mM) was then added where indicated, and during rounds of mutagenesis, 5 mM arabinose was also added. Cultures were then infected with 10^4^–10^6^ pfu/ml M13ΔgIII:*Ma*PylRS. The infected culture grew overnight at 37°C, and phages were harvested the next morning. In later rounds of higher stringency positive selection, plasmids pJT018 (gIII_2xTAG_ *Ma*tRNA^Pyl^_(6)_) and pJT019 (gIII_3xTAG_ *Ma*tRNA^Pyl^_(6)_) were used in place of pJT017.

Negative selection was carried out using the negative selection plasmids pJF011 (T7RNAP_2xTAG_ *Ma*tRNA^Pyl^_(6)_) and pDB016 (carrying gIII under a T7 promoter). 30 ml cultures were grown in 2xYT media, and at an OD_600_ of 0.4–0.6, the cells were infected with 10^4^–10^6^ pfu/ml of phage. The culture grew overnight at 37°C, and phages were harvested the following morning.

Phage titers were performed at the conclusion of every generation as a checkpoint to ensure that phage propagation remained robust and selective for BocK. Collected phage samples were serially diluted and plated on LB-top agar plates containing *E. coli* S1059 cells. 5 µL of each serial dilution were spotted on the plates, and the pfu/mL for each phage lineage +/− BocK was calculated.

### *In vivo* Fluorescence Assays

Electrocompetent *E. coli* DH10B cells were transformed with a pMW plasmid encoding the PylRS variant, and a pBAD plasmid encoding sfGFP 2TAG and *Ma*tRNA^Pyl^. Variations of the two plasmids were cloned and used where indicated. Colonies of freshly transformed DH10B cells harboring the indicated plasmids were picked and grown at 37°C overnight in 5 ml of LB media supplemented with appropriate antibiotics. 2 µL of the overnight cultures were diluted into 150 µL LB supplemented with 500 µM IPTG, 0.2% arabinose, appropriate antibiotics, and 1 mM of the indicated ncAA. Inoculated cultures were grown shaking at 37°C in a BioTek Synergy plate reader. Fluorescence intensity (*λ*_ex_ = 485nm, *λ*_em_ = 528 nm) and OD_600_ were measured every 15 min, and fluorescence/OD_600_ was calculated using the 12-h time point. Fluorescence/OD_600_ measurements and standard deviations were calculated based on data collected from three biological and two technical replicates.

For the dual ncAA incorporation assay, a three-plasmid system was used: 1) pMW AzFRS.2. t1, 2) pULTRA encoding the PylRS variant and the ochre-suppressing mutant tRNA *Ma*tRNA^Pyl^_(6)/UUA_, and 3) pBAD sfGFP 2TAG 149TAA *M. jannaschii* tRNA^Tyr^_CUA_. The plasmids were co-transformed into electrocompetent *E. coli* DH10B cells. The rest of the procedure follows the above protocol.

### Protein Expression and Purification

Electrocompetent DH10B cells were co-transformed with pBAD sfGFP 2TAG *Ma*tRNA^Pyl^ and pMW *Ma*PylRS or PylRS_opt_. Fresh colonies were used to inoculate 20 ml LB media supplemented with 0.2% arabinose, 500 µM IPTG, 1 mM BocK, 25 μg/ml spectinomycin, and 100 μg/ml ampicillin. Cultures were grown shaking at 37°C overnight. The following morning, the cultures were centrifuged at 4,000 × g for 10 min, and the pellets were resuspended in 1X BugBuster protein extraction reagent (Millipore-Sigma). The lysate was clarified by centrifugation (15,000 × g for 20 min) and the cleared lysate was incubated with nickel-NTA agarose beads for 20 min. The beads were washed five times with wash buffer (50 mM Tris pH 8.0, 50 mM NaCl, 10 mM imidazole), and eluted with elution buffer (50 mM Tris pH 8.0, 50 mM NaCl, 250 mM imidazole. The purified proteins were concentrated and buffer-exchanged into 25 mM Tris pH 8.0, 25 mM NaCl using an Amicon Ultra Centrifugal Filters (10 kDa MWCO).

### Mass Spectroscopy

Mass spectroscopy was performed by Bioinformatics Solutions Inc. in Waterloo, ON, Canada. LC-MS analysis of DTT reduced samples were performed on a Thermo Scientific Orbitrap Exploris 240 mass spectrometer, equipped with a heated electrospray ionization source (H-ESI) in positive ion mode with a Thermo Fisher Ultimate 3000 RSLCnano HPLC System. On the H-ESI source, sheath gas was set to 2 arbitrary units (arb), and auxiliary gas was set to 6 arb. The ion transfer tube was set at 275°C. The vaporizer temp was at 200°C. The sample was analyzed on a MAbPac RP, 4 µM, 3.0 × 50 mm analytical column (ThermoFisher, San Jose, CA, United States), held at 60°C. The protein was eluted at a rate of 500 μL/min for a 10-min gradient, where 0–7 min: 10–70% acetonitrile +0.1% formic acid; 7–8.2 min: 95% acetonitrile +0.1% formic acid, 8.2–10 min: 20% acetonitrile +0.1% formic acid. MS spectra were acquired using full scans at 15,000 resolution in the orbitrap within a range of 700–2,200 m/z. The maximum injection time was set at auto with a standard AGC target. Ten micro scans were employed, and the RF lens was set to 60%. 15 V of insource CID was applied. Thermo BioPharma Finder 4.1 was used for intact mass deconvolution and peak identification.

### Chloramphenicol Acetyltransferase Assay

Electrocompetent DH10B cells were transformed with a pMW plasmid encoding the PylRS variant, and a pCAM plasmid encoding chloramphenicol acetyltransferase 112TAG and *Ma*tRNA^Pyl^. Colonies of freshly transformed DH10B cells harboring the indicated plasmids were picked and grown at 37°C overnight in 5 ml of LB media supplemented with appropriate antibiotics. The overnight cultures were serially diluted, and 2 µL of each dilution were spotted onto LB-agar plates supplemented with the appropriate antibiotics for plasmid maintenance, 100 µM IPTG, 1 mM of the ncAA where indicated, and either 100 (PrK plates) or 200 (BocK and ALocK) µg/mL chloramphenicol. The spotted plates were grown at 37°C overnight. Images were taken after 16 h of incubation.

## Results

### Developing a PANCE Protocol to Evolve *Ma*PylRS

**FIGURE 1:**
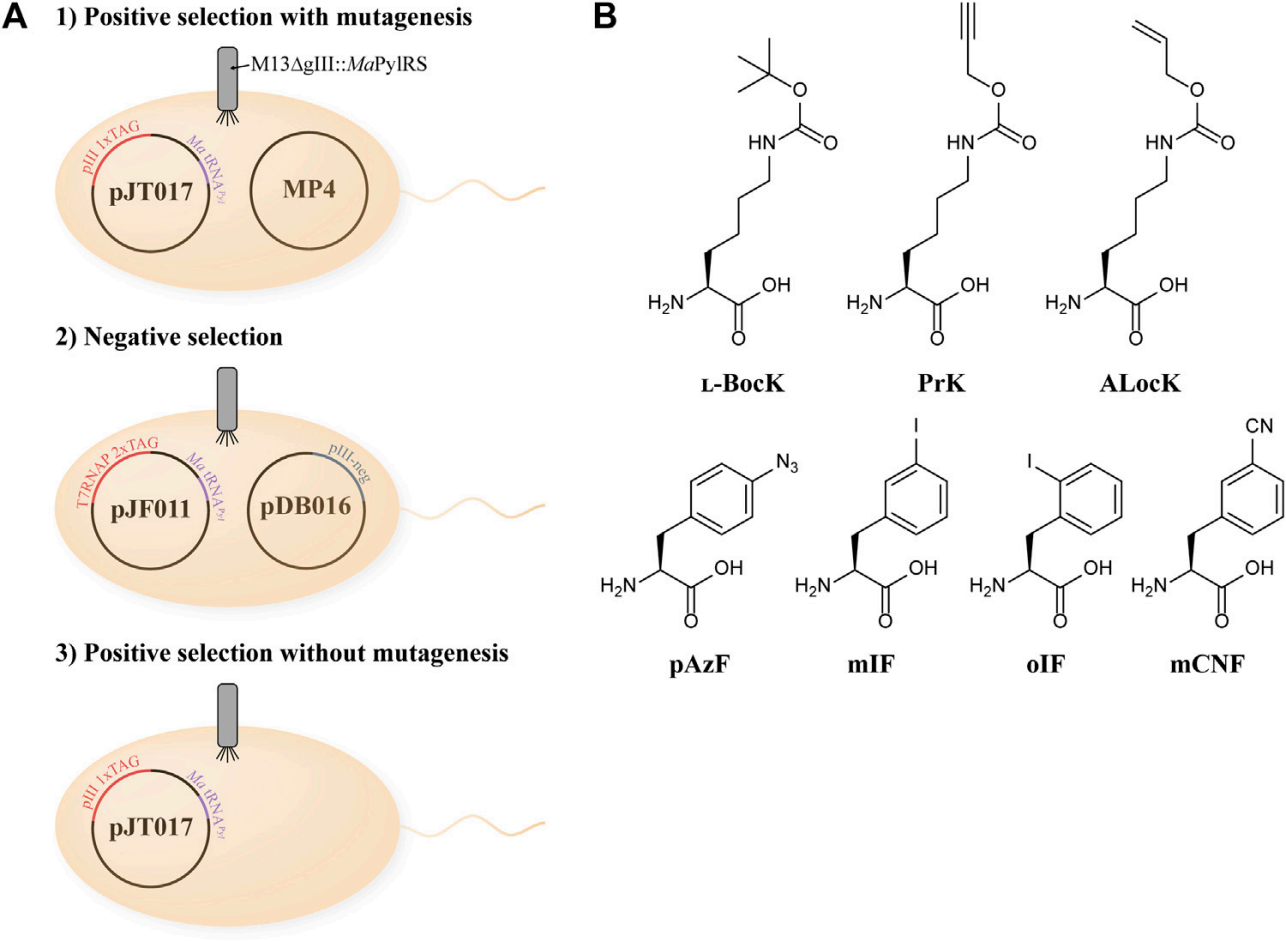
**(A)** Overview of the PANCE experimental design. **(B)** Structures of ncAAs used in this study.

To evolve a hyperactive variant of *Ma*PylRS that also discriminates against canonical amino acids, we adapted a variation of the previously described PANCE system (Figure 1A) (Suzuki et al., 2017). For each generation of phage evolution, we performed a three-step selection process: positive selection with mutagenesis, followed by negative selection, and finally positive selection without mutagenesis. In the first step, *E. coli* S1030 cells are transformed with the MP4 mutagenesis plasmid and an accessory plasmid (AP), pJT017 (Badran and Liu, 2015; Bryson et al., 2017). The AP encodes the essential phage protein pIII with one to three TAG codons, as well as *Ma*tRNA^Pyl^_(6)_, an engineered tRNA^Pyl^ variant that is orthogonal to the *M. mazei* PylRS/tRNA^Pyl^ system (Supplementary Figure S1) (Willis and Chin, 2018). We utilized *Ma*tRNA^Pyl^_(6)_ in our PANCE system to determine if the tRNA binding domain of *Ma*PylRS would evolve a greater affinity for *Ma*tRNA^Pyl^_(6)_. The transformed *E. coli* S1030 cells are grown to mid-log phase, supplemented with BocK (Figure 1B) and arabinose, and infected by the phage M13 ΔgIII::*Ma*PylRS. Phage propagation is dependent on successful aminoacylation of tRNA^Pyl^ by *Ma*PylRS and subsequent suppression of the TAG codon(s) in gIII (the gene encoding pIII), thus enabling selection for phages carrying the most active PylRS variants.

To avoid the unwanted evolution of a promiscuous synthetase that is active towards canonical amino acids, after each round of positive selection with mutagenesis, we performed negative selection in the absence of BocK. Originally developed as an additional selection mechanism in the continuous directed evolution technique PACE, negative selection is highly effective in maintaining the amino acid selectivity of evolving aminoacyl-tRNA synthetases (Carlson et al., 2014; Bryson et al., 2017). The negative selection system relies on the production of pIII-neg, a dominant-negative version of pIII. Production of pIII-neg inhibits phage propagation, thus depleting the population of phages carrying PylRS mutants that are active towards canonical amino acids and can produce pIII-neg in the absence of BocK. In our pilot experiments to optimize the PANCE system for evolving *Ma*PylRS, we found that without negative selection, significant phage propagation occurs in the absence of BocK after consecutive generations of positive selection with mutagenesis (Supplementary Figure S2). This indicates that *Ma*PylRS is sensitive to mutations that enable activity towards a canonical amino acid, most likely Phe. This is predictable, as *M. mazei* PylRS has been shown to readily incorporate Phe when the critical active site residues N346 (N166 in *M. alvus*) and C348 (V168) are mutated (Wang et al., 2011). We therefore adopted the negative selection step to maintain the selectivity of evolving *Ma*PylRS variants.

**FIGURE 2:**
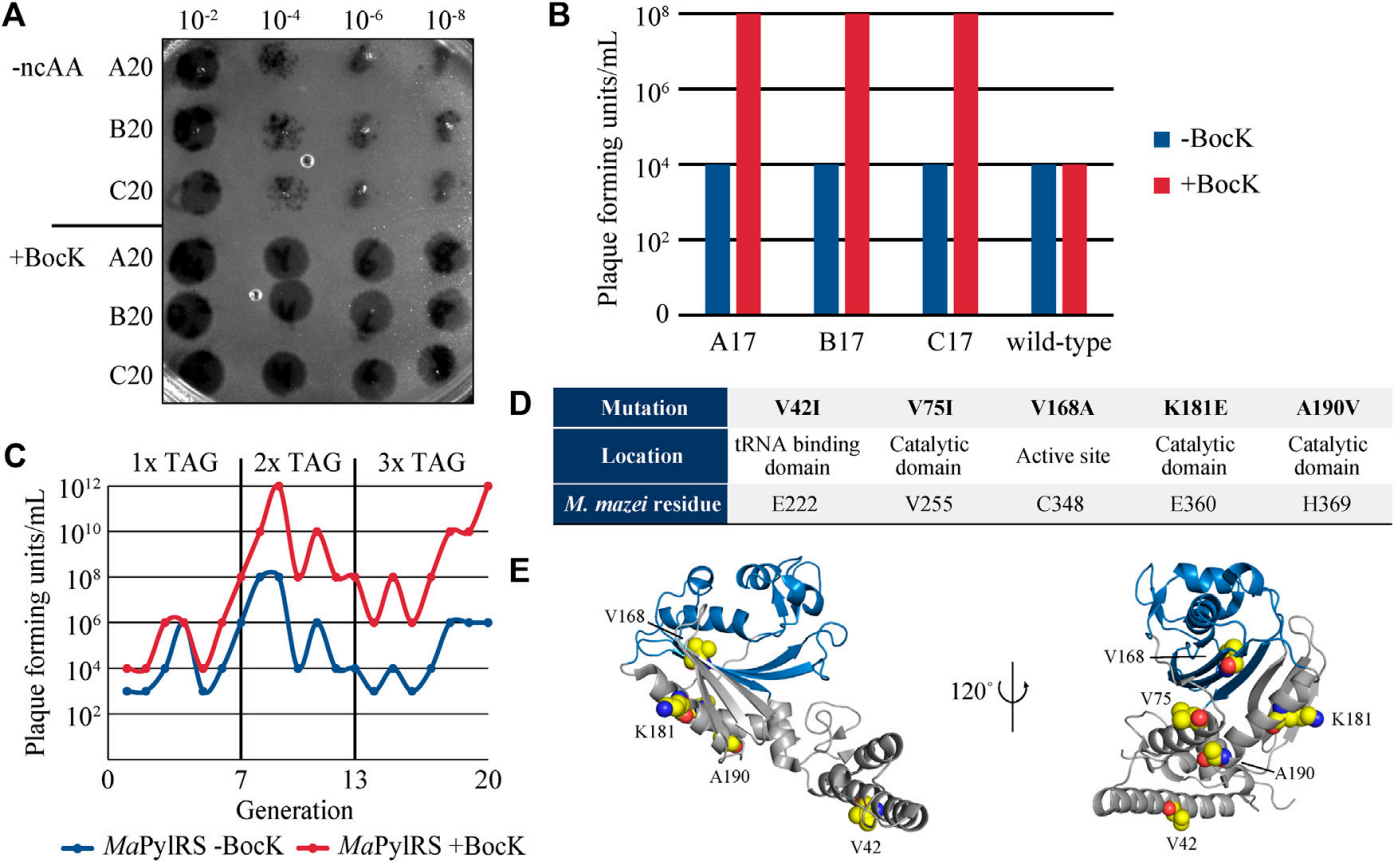
**(A)** Phage titers of the 20th generation of evolved M13 *Ma*PylRS. The numbers above the image indicate the dilution factor. **(B)** Measurements of phage titers comparing the 17th generation of evolved phages and the ancestral generation zero encoding wild-type *Ma*PylRS. Cells were infected and grown in the presence and absence of BocK. **(C)** Summary of phage titers over 20 generations, in the presence and absence of BocK. **(D)** Mutations to *Ma*PylRS evolved through PANCE, as well as their location in the protein and the corresponding *M. mazei* residue as determined by multiple sequence alignment. **(E)** Crystal structure of *Ma*PylRS. Residues that were mutated during PANCE are highlighted as spheres (PDB ID: 6JP2).

As a final selection step, the surviving phage population isolated from the prior negative selection was passed through a round of positive selection without mutagenesis. This final step amplifies phages that survived negative selection and are still active towards BocK without the added stringency of mutagenesis. We performed this selection in both the presence and absence of BocK, and used the phages isolated from these two conditions as a checkpoint to verify that evolution was proceeding successfully. We measured the phage titers of both conditions to ensure that phage propagation remained robust in the presence of BocK, and low when BocK was not added to the media (Figure 2A). The significant difference between the phage titers indicates that promiscuous variants are not persisting through the negative selection step, and variants that are active towards BocK are indeed being selected. The phage population recovered from this final round of positive selection in the presence of BocK was carried over to the next generation of selection, and the three-step cycle was repeated.

### Directed Evolution of *Ma*PylRS

We performed a total of 20 generations of directed evolution and maintained three independent lineages of evolved phages. The stringency of selection was increased as evolution proceeded. Initially, gIII carried a single in-frame TAG codon to be suppressed by *Ma*PylRS/tRNA^Pyl^_(6)_. After 7 generations of selection, we increased the stringency to two TAG codons in gIII. The evolved phage population efficiently propagated in this higher stringency selection when cultures were supplemented with BocK, while maintaining low levels of propagation in the absence of BocK. After 6 rounds of selection with two TAG codons, we again increased the stringency to three TAG codons. Even at this highest level of stringency, phage propagation remained robust and highly selective for BocK. At this stage, we compared the propagation of our evolved phage lineages to that of the ancestral variant (generation zero) that encodes wild-type *Ma*PylRS. We measured propagation under the high-stringency three TAG codon system in the absence of mutagenesis. The phage titer indicated that the evolved phage lineages propagated far more efficiently than generation zero in the presence of BocK, suggesting that more active variants of PylRS had emerged (Figure 2B).

Throughout the course of the directed evolution process, phage titers at the end of each generation consistently showed greater phage propagation when BocK was present in the media (Figure 2C). After the 20th round of evolution, several plaques from each evolved lineage were sequenced to determine the identity of PylRS mutations. Five mutations were identified, with very little crossover between each of the three independent lineages (Figure 2D). The most frequently occurring mutations that arose from our system were V42I, V75I, V168A, K181E, and A190V. V42I and K181E were found as concomitant mutations, while the rest of the variants were point mutations. V168A is the only active site mutation that was observed. This mutation has been identified previously and exploited for its role in substrate specificity (Wang et al., 2011; Wang et al., 2012; Wan et al., 2014; Seki et al., 2020). As such, we hypothesized that this mutation to the smaller alanine residue may confer altered substrate specificity favoring larger ncAAs such as BocK. V75I, K181E, and A190V are located in the catalytic domain just outside of the active site, while V42I is found in the tRNA binding domain (Figure 2E). Interestingly, multiple sequence alignment shows that the K181E mutation matches the sequence of several PylRS homologs that also encode Glu at this position (E360 in *M. mazei* PylRS) (Supplementary Figure S3) (Yanagisawa et al., 2008). To the best of our knowledge, aside from V168, none of these residues have previously been identified or targeted for engineering in *Ma*PylRS or any other PylRS homologs.

### *Ma*PylRS_evo_ Variants Are Highly Active and Selective for ncAAs *in vivo*

**FIGURE 3:**
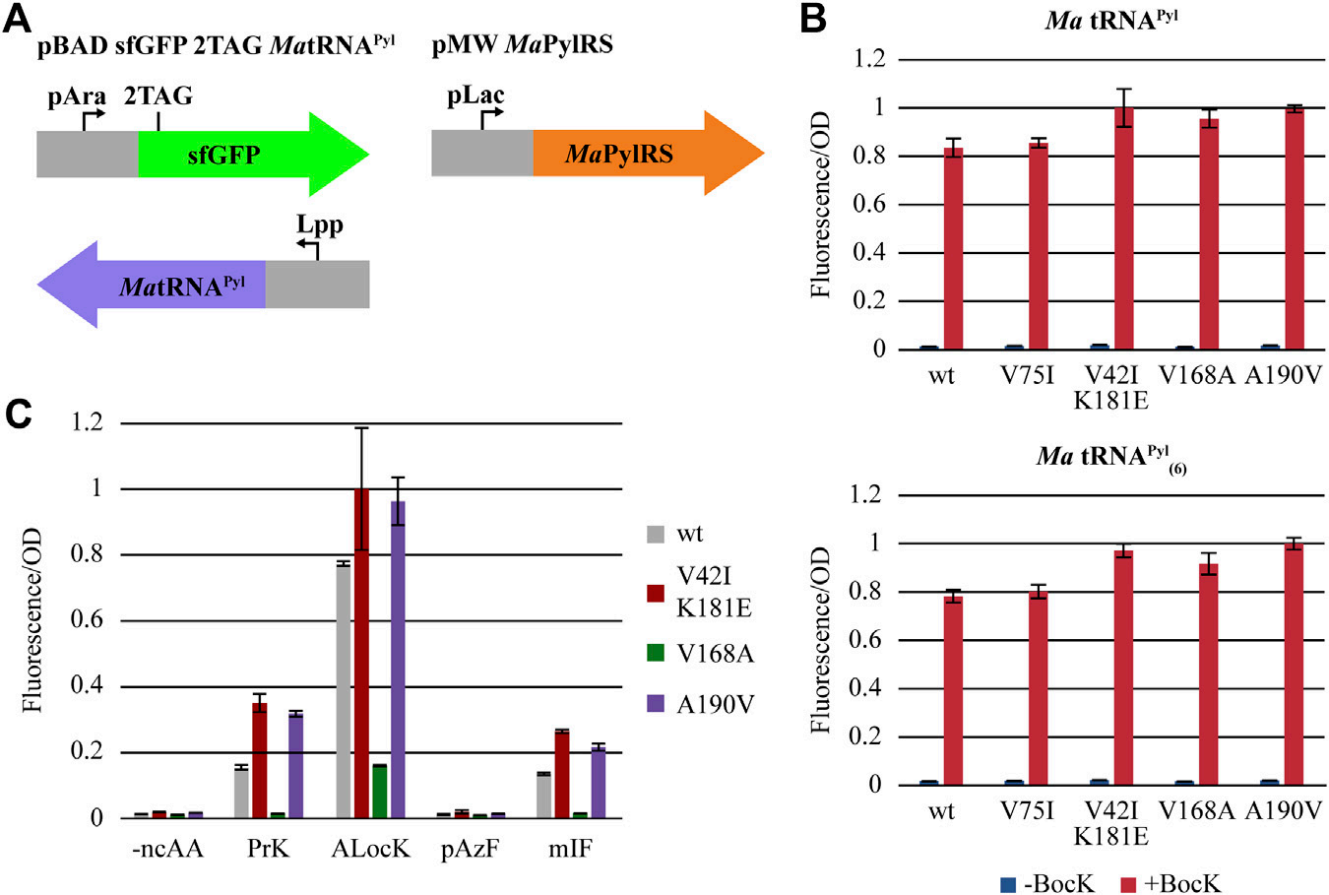
**(A)** Overview of the *in vivo* fluorescence reporter system. **(B)** Initial fluorescence screening of PylRS mutants, using wild-type *Ma*tRNA^Pyl^ (left) and *Ma*tRNA^Pyl^_(6)_ (right). **(C)** Fluorescence measurements of the most active mutants and a selection of ncAAs. Error bars indicate the standard deviation for each condition (*n* = 6).

To test the activity of our evolved *Ma*PylRS mutants, we cloned the mutant genes into a pMW expression vector and co-transformed them along with a pBAD reporter plasmid carrying sfGFP (2TAG) and tRNA^Pyl^ into *E. coli* DH10B. Read-through of the TAG codon at position 2 is dependent on PylRS aminoacylating its cognate tRNA^Pyl^ with a ncAA. Aminoacyl-tRNA^Pyl^ is then utilized in translation to suppress TAG and incorporate the ncAA into the protein (Figure 3A). Thus, fluorescence from sfGFP production can be used to measure the activity of PylRS. We measured the fluorescence/OD_600_ of cells expressing our PylRS variants and compared this to those expressing wild-type *Ma*PylRS, both in the presence and absence of BocK (Figure 3B). The results show that each of the four mutant constructs as well as wild-type *Ma*PylRS are highly active and selective for BocK. The lack of background activity in the absence of a ncAA for all four mutants substantiates the importance of the negative selection step in our PANCE system. We tested the activity of the variants using both wild-type *Ma*tRNA^Pyl^ and *Ma*tRNA^Pyl^_(6)_. The data indicate that both *Ma*tRNA^Pyl^ and *Ma*tRNA^Pyl^_(6)_ are efficiently charged with BocK by all four mutant variants and wild-type *Ma*PylRS. The activity of the evolved variants towards *Ma*tRNA^Pyl^_(6)_ is consistent with that of wild-type *Ma*PylRS, suggesting that the variants did not evolve enhanced recognition of *Ma*tRNA^Pyl^_(6)_. However, compared to wild-type *Ma*PylRS, the activity towards BocK is improved for three of the four mutants, with the exception being V75I which is approximately equal to wild-type.

We hypothesized that in addition to having robust activity towards the directed evolution substrate BocK, our mutants may also have improved activity towards other Lys- and Phe-ncAAs. We screened four additional ncAAs: *N* ^ε^-propargyloxycarbonyl-l-lysine (PrK), *N* ^ε^-allyloxycarbonyl-l-lysine (ALocK), 4-azido-l-phenylalanine (pAzF), and 3-iodo-l-phenylalanine (mIF) (Figure 3C). The most active variants, V42I/K181E and A190V, have dramatically improved activity towards PrK, ALocK, and mIF. Interestingly, the activity of the active-site mutant V168A is significantly decreased to all the ncAAs we tested except BocK, highlighting the previously established role of this mutation in governing the size and selectivity of the PylRS active site (Wang et al., 2011; Wang et al., 2012; Wan et al., 2014; Seki et al., 2020). The activity of V75I is moderately higher towards PrK compared to the most active variants, and we omitted V75I from further screening. None of the mutants have significant activity towards the *para*-substituted phenylalanine derivative pAzF. As such, we hypothesized that like *Ma*PylRS, the evolved PylRS variants should also be orthogonal to the *M. jannaschii*-derived AzFRS.2. t1, enabling them to be used jointly to incorporate multiple, distinct ncAAs into a single protein (Amiram et al., 2015; Tharp et al., 2021).

### Combining the Most Active PANCE Mutations to Create PylRS_opt_

**FIGURE 4:**
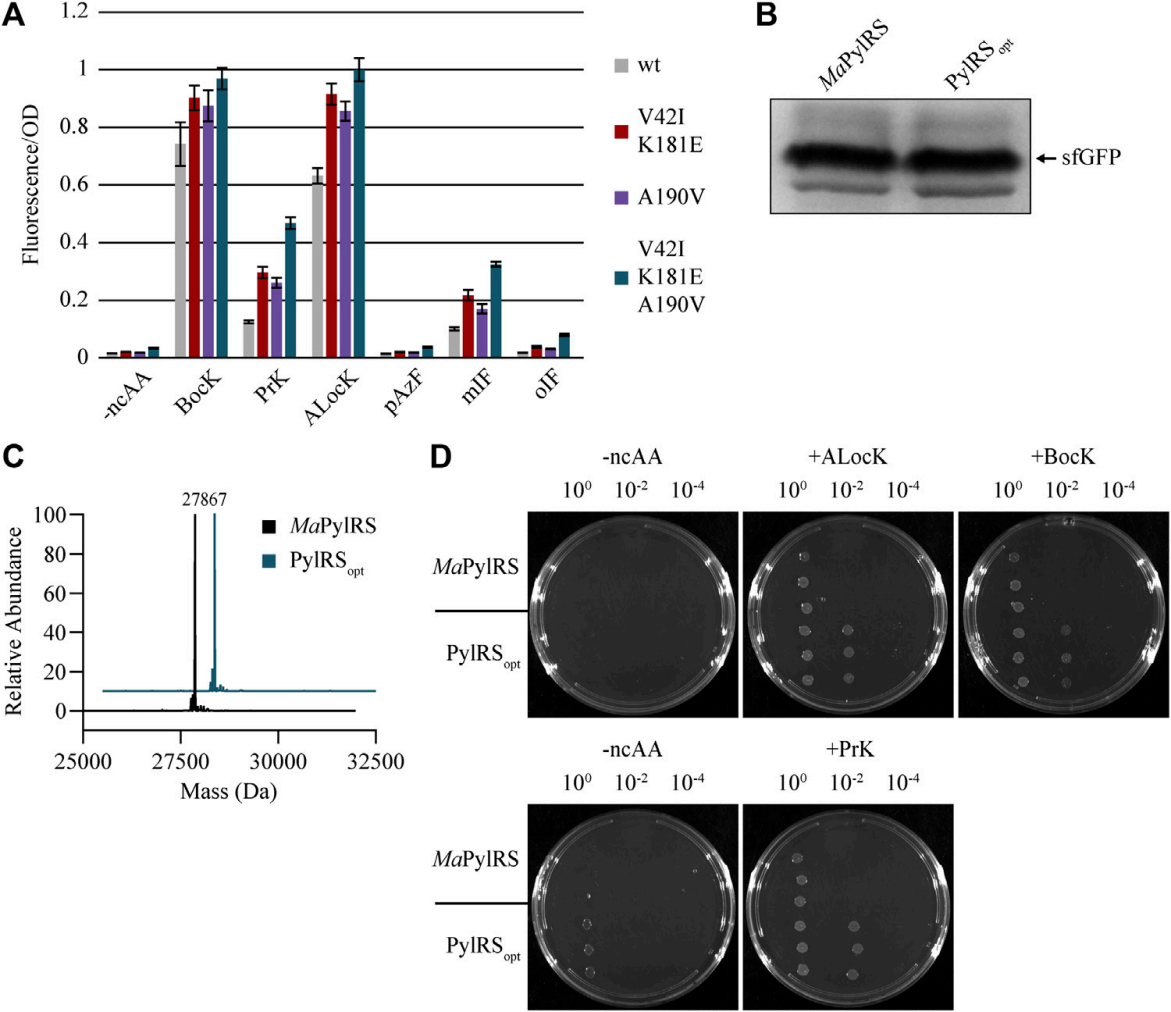
**(A)** *In vivo* fluorescence assay measuring the activity of wild-type and evolved *Ma*PylRS variants and the combined mutant PylRS_opt_. **(B)** SDS gel showing relative protein yields of sfGFP-2BocK from cells expressing either *Ma*PylRS or PylRS_opt_. **(C)** LC-MS of the samples shown in Figure 4B. Peaks were detected for sfGFP-2BocK (theoretical mass = 27,869 Da) purified from cells expressing either *Ma*PylRS or PylRS_opt_. **(D)** Chloramphenicol acetyltransferase assay. The top and bottom row of plates are supplemented with 200 μg/ml and 100 μg/ml chloramphenicol, respectively.

We suspected that combining the mutations that conferred the greatest increases in activity may result in an additive effect, yielding a hyperactive, ncAA-specific PylRS variant. Based on our results from the initial fluorescence experiments, we cloned a *Ma*PylRS variant containing the mutations V42I, K181E, and A190V, and named the construct PylRS_opt_. We tested the activity of *Ma*PylRS, PylRS_V42I/K181E_, PylRS_A190V_, and PylRS_opt_ towards several ncAAs, and found that the activity of PylRS_opt_ was higher than both wild-type and the individual mutants for every ncAA we tested (Figure 4A). Notably, background fluorescence in the absence of ncAA remains close to basal levels for PylRS_opt_. Like *Ma*PylRS, PylRS_opt_ is highly selective for ncAAs over canonical amino acids. To support this, we co-transformed DH10B cells with sfGFP 2TAG and either *Ma*PylRS or PylRS_opt_. The cells were supplemented with 1 mM BocK, and sfGFP was purified. The yield of sfGFP was robust at approximately 20 mg/L in cells expressing either *Ma*PylRS or PylRS_opt_ (Figure 4B). We analyzed these samples by intact mass spectroscopy, which confirmed that BocK is solely present at position 2 of the protein (Figure 4C).

To gain further insight into the robustness of the activity of PylRS_opt_, we titrated BocK, ALocK, and PrK and measured the fluorescence in cells expressing either *Ma*PylRS or PylRS_opt_ (Supplementary Figure S4). We tested concentrations as low as 62.5 µM, which is slightly above the K_m_ that was reported for *M. mazei* PylRS and its natural substrate, pyrrolysine (Guo et al., 2014). We found that the activity of PylRS_opt_ is significantly greater than *Ma*PylRS at all ncAA concentrations tested.

To validate our *in vivo* fluorescence data, we implemented a chloramphenicol acetyltransferase assay as a second reporter system. DH10B cells expressing either *Ma*PylRS or PylRS_opt_, *Ma*tRNA^Pyl^, and the chloramphenicol acetyltransferase gene with a single in-frame TAG codon were challenged to grow on plates containing bacteriostatic levels of chloramphenicol in the presence and absence of BocK, PrK, and ALocK (Figure 4D). The results corroborated our observations from the fluorescence data. Upon diluting saturated overnight cultures 1/100, cells expressing *Ma*PylRS are unable to grow on chloramphenicol plates regardless of the presence or absence of a ncAA. However, under these same conditions, PylRS_opt_ supports strong colony growth in the presence of BocK, PrK, or ALocK. Neither enzyme supports growth on 200 μg/ml chloramphenicol plates in the absence of a ncAA. We note, however, that on the less stringent 100 μg/ml chloramphenicol plates, spotting undiluted cells expressing PylRS_opt_ enables slow growth in the absence of a ncAA, whereas the same cells expressing *Ma*PylRS cells are unable to grow under these conditions. This phenomenon is not observed at 200 μg/ml chloramphenicol, where growth is only apparent for PylRS_opt_ in the presence of 1 mM BocK or ALocK. These results confirm the previous observation that PylRS_opt_ is more active than *Ma*PylRS towards BocK, ALocK, and PrK, with the caveat that under low stringency conditions, PylRS_opt_ can apparently incorporate a canonical amino acid in the absence of a ncAA and facilitate modest colony growth.

### Inserting PylRS_opt_ Mutations Into *Ma*PylRS N166S

**FIGURE 5:**
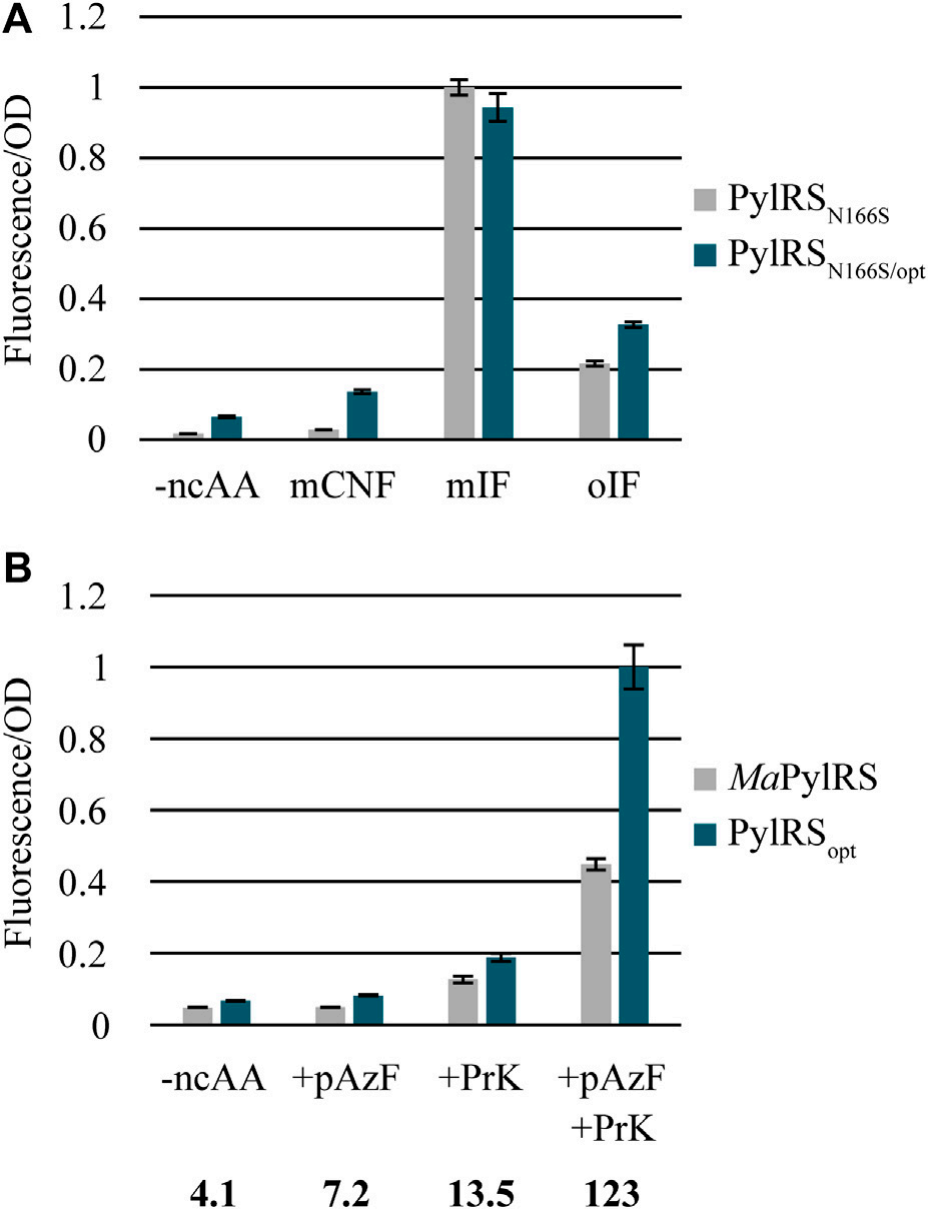
**(A)** Combining PylRS_opt_ mutations with N166S improves incorporation of mCNF, oIF. **(B)** Dual ncAA incorporation in a reporter protein using AzFRS.2. t1 and either *Ma*PylRS or PylRS_opt_. The numbers below each condition indicate the percent increase of fluorescence/OD in cells expressing PylRS_opt_ relative to those expressing *Ma*PylRS.

Next, we sought to determine whether the mutations found in PylRS_opt_ can improve the activity of a previously engineered variant of *Ma*PylRS that readily incorporates *meta-* and *ortho-*substituted Phe (Tharp et al., 2021). The highly conserved active site residue N166 (N346 in *M. mazei* PylRS) plays a critical role as the “gatekeeper residue” of the PylRS active site, as the amide nitrogen is involved in the recognition of pyrrolysine and its analogs (Yanagisawa et al., 2008; Wang et al., 2011; Wang et al., 2012). Mutating N166 alters the substrate specificity of *Ma*PylRS, effectively eliminating recognition of lysine-derived ncAAs while greatly increasing its activity towards several phenylalanine derivatives. *Ma*PylRS_N166S_ was recently shown to aminoacylate a variety of *meta-* and *ortho-*substituted phenylalanine derivatives (Tharp et al., 2021). Thus, we created PylRS_N166S/opt_ and compared its activity to *Ma*PylRS_N166S_ to assess whether combining the PylRS_opt_ mutations with N166S can enhance its activity towards Phe derivatives without compromising its selectivity (Figure 5A). The results show that PylRS_N166S/opt_ has increased activity towards mCNF and oIF, which are both difficult substrates for *Ma*PylRS_N166S_. However, the PylRS_opt_ mutations do not improve activity towards the well-recognized substrate mIF. As *Ma*PylRS_N166S_ is already highly active towards mIF, the lack of improvement in activity towards this substrate is predictable. It should be noted that the background activity of PylRS_N166S/opt_ is also elevated in the absence of a ncAA. It is plausible that the canonical amino acid may be sufficiently outcompeted when a recognized ncAA is present, as is the case with PylRS_opt_ in the presence of BocK. Overall, the data indicate that the mutations in PylRS_opt_ improve the activity of *Ma*PylRS_N166S_ towards difficult Phe-ncAAs. However, elevated background activity is also apparent for this PylRS variant and may be a drawback for applications that require a homogeneous protein sample. Lower background levels may be attainable when combining the PylRS_opt_ mutations with other active site mutants variants, such as those reported previously for incorporating bulky Lys-ncAAs (Seki et al., 2020).

### PylRS_opt_ Mutations Improve the Efficiency of Dual ncAA Incorporation

As a final assessment of the usefulness of PylRS_opt_, we tested whether the enhanced activity and substrate specificity of PylRS_opt_ could be used to improve the incorporation of two distinct ncAAs into a single protein. Translation efficiency drops dramatically when two or more distinct ncAAs are to be inserted into a protein, a problem that is exacerbated when aminoacylation of either of the ncAAs is particularly challenging (Hoesl and Budisa, 2011). Thus, improved activity is crucial to attaining substantial yields of proteins containing multiple ncAAs. To investigate the applicability of PylRS_opt_ for improving dual incorporation of difficult ncAAs, we cloned a system to measure the production of sfGFP featuring two in-frame stop codons: 2TAG and 149TAA. We utilized the *M. jannaschii* TyrRS variant AzFRS.2. t1 and its cognate MjtRNA^Tyr^_CUA_ to incorporate pAzF, and either *Ma*PylRS or PylRS_opt_ along with *Ma*tRNA^Pyl^_(6)/UUA_ to incorporate PrK. Importantly, PylRS does not discriminate against the anticodon of tRNA^Pyl^ (Ambrogelly et al., 2007), and tRNA^Pyl^_UUA_ does not suppress UAG codons (Odoi et al., 2013), thus enabling PylRS/tRNA^Pyl^_UUA_ to function as an orthogonal pair with the *Mj*TyrRS/tRNA^Tyr^_CUA_ system (Wan et al., 2010). When cultures were supplemented with both pAzF and PrK, we observed over 120% increased fluorescence in cells expressing PylRS_opt_ as opposed to *Ma*PylRS (Figure 5B). Background activity in the absence of one or both ncAAs was comparable for PylRS and PylRS_opt_. Interestingly, low levels of expression were seen when PrK was present but pAzF was omitted, consistent with earlier observations of the PylRS/*Mj*TyrRS dual ncAA incorporation system (Wan et al., 2010; Chatterjee et al., 2013). As has been suggested previously, this artifact is likely due to low-level recognition of canonical amino acids by AzFRS.2. t1 in the absence of pAzF. Ultimately, utilizing PylRS_opt_ to incorporate a second ncAA into a protein leads to over a two-fold improvement in the apparent protein yield compared to wild-type *Ma*PylRS. PylRS_opt_ should therefore be quite useful in applications requiring site-specific incorporation of multiple reactive moieties in a single protein, such as bioorthogonal click chemistry (Neumann et al., 2010; Wan et al., 2010).

## Discussion

We have shown that the PANCE system can be adjusted to mitigate the emergence of promiscuous PylRS variants by integrating a negative selection step into the procedure. Alternating positive and negative selection while gradually increasing the stringency of positive selection enables the emergence of highly selective, hyperactive variants of *Ma*PylRS. Screening the activity of *Ma*PylRS mutants that arose from PANCE led us to the identification of PylRS_opt_, a novel PylRS construct. Despite having no modifications to its active site, PylRS_opt_ profiles as a valuable genetic code expansion tool that is highly active towards several Lys- and Phe-ncAAs while maintaining excellent selectivity in discriminating against canonical amino acids. All but one of the mutations that arose from our directed evolution process were previously unidentified, and the mechanism by which these mutations enhance PylRS activity is unclear. Nevertheless, the identification of several residues outside of the active site underscores the power of impartial mutagenesis and selection, as it is unlikely that these residues would have otherwise been targeted for engineering.

Our findings of impactful mutations outside of the active site are consistent with previously reported directed evolution experiments on chPylRS (Bryson et al., 2017; Suzuki et al., 2017). In these studies, increased PylRS activity is proposed to stem from improved binding to the cognate tRNA. In *Ma*PylRS, K181 and A190 are located on an α-helix just outside of the active site (Seki et al., 2020). It is plausible that the mutations we observed may create conformational changes that alter the size, shape, or flexibility of the catalytic core. Conversely, V42 is located in the tRNA binding domain. This mutation is unlikely to impact ncAA binding but may instead facilitate a change in tRNA interaction. In the *D. hafniense* PylRS/tRNA^Pyl^ complex, the corresponding aligned residue Q52 is located in the α-helix of tRNA binding domain 1 that serves as a binding surface for the core of tRNA^Pyl^ (Nozawa et al., 2009). Thus, it is possible that mutation of V42 may alter the binding of *Ma*PylRS to tRNA^Pyl^, although it is unclear how the conserved V42I mutation may impact the overall structure of the domain. It is also possible that V42I is an innocuous mutation and K181E is instead the sole driving force behind the activity of this PylRS variant, as we did not test the activity of each of the two mutants independently. Accordingly, the opposite may hold true as well.

Our directed evolution system led to the emergence of several PylRS variants that are active towards a variety of ncAAs. The lone exception to this observation is the active site mutant V168A, which is capable of efficiently incorporating BocK but discriminates against all the other Lys- and Phe-derivatives we tested. V168 (C348 in the *M. mazei* sequence) is associated with the size of the active site binding pocket and is positioned near the *N* ^ε^-substituent of pyrrolysine and its analogs (Kavran et al., 2007; Seki et al., 2020). Thus, it stands to reason that substituting the smaller alanine at this position enlarges the binding pocket. This may then allow the bulky BocK to maintain contacts with the active site while simultaneously hindering the recognition of smaller canonical and noncanonical amino acids. Conversely, mutating V168 to a larger residue may improve the recognition of smaller ncAAs as well as canonical phenylalanine, as this was observed in a mutational analysis of *M. mazei* PylRS (Wang et al., 2011).

Combining the most active PANCE mutations to generate PylRS_opt_ resulted in an additive effect: the activity of the combined mutant is demonstrably higher than its predecessors. The enhanced activity of PylRS_opt_ is particularly notable for ncAAs that *Ma*PylRS is relatively weakly active towards, such as PrK. While the greatest activity increases are observed when using weaker substrates, PylRS_opt_ is also more active towards well-recognized ncAAs such as BocK and ALocK. Further, background activity in the absence of a ncAA is not significantly altered by the mutations in PylRS_opt_. We suspect that the activity increase seen in PylRS_opt_ is generalizable and not substrate-specific, as we observed enhanced activity for PylRS_opt_ towards nearly every substrate that the wild-type enzyme is also active towards. Utilizing PylRS_opt_ in future studies to incorporate additional ncAAs should validate this hypothesis. Because the PylRS_opt_ mutations are located outside of the active site, these mutations should theoretically be generalizable like those found in the evolved chPylRS variant (Bryson et al., 2017). Although the results with *Ma*PylRS_N166S/opt_ were somewhat mixed, future studies may reveal other engineered PylRS variants that are improved by the PylRS_opt_ mutations. Finally, we have shown that PylRS_opt_ is orthogonal with the *Mj*TyrRS/tRNA^Tyr^ system and enables a sharp increase in the production of a reporter protein with two distinct ncAAs. It is likely that when used with tRNA^Pyl^_(6)_, PylRS_opt_ is orthogonal with the *M. mazei* PylRS/tRNA^Pyl^ system, as is the case with *Ma*PylRS/tRNA^Pyl^_(6)_ (Willis and Chin, 2018). Thus, incorporation of three distinct ncAAs should also be improved by PylRS_opt_ when used in combination with the other two orthogonal systems as previously described (Tharp et al., 2021).

In summary, directed evolution facilitated the development of a hyperactive and highly selective genetic code expansion tool PylRS_opt_, which should be useful for a wide variety of applications moving forward.

## Data Availability

The raw data supporting the conclusion of this article will be made available by the authors, without undue reservation.
