# Supplementary material for: Directed Evolution of Methanomethylophilus alvus Pyrrolysyl-tRNA Synthetase Generates a Hyperactive and Highly Selective Variant
Source: Front Mol Biosci. 2022 Mar 9;9:850613. doi: 10.3389/fmolb.2022.850613 (PMC8965510; doi:10.3389/fmolb.2022.850613)
Supplement: Supplementary file 1 [file DataSheet1.PDF]

# *Supplementary Material*

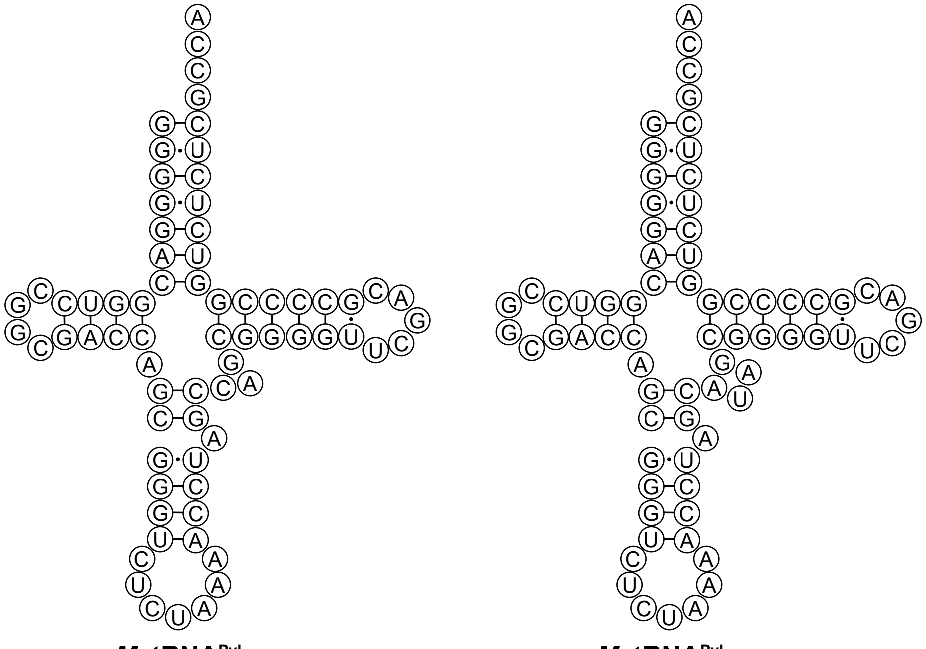

***Ma*tRNA$^{Pyl}$**    ***Ma*tRNA$^{Pyl}_{(6)}$**

**Supplementary Figure 1.** Cloverleaf structures of *Ma*tRNA$^{Pyl}$ and *Ma*tRNA$^{Pyl}_{(6)}$.

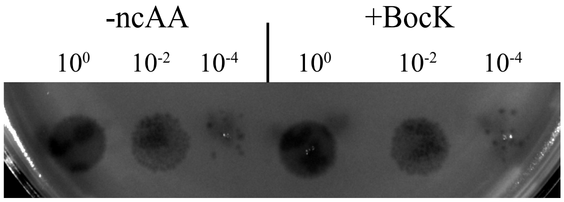


**Supplementary Figure 2.** Consecutive generations of positive selection with mutagenesis leads to propagation in the absence of ncAA. The numbers above each spot on the plate indicate the dilution factor of the titer.

```
    M. alvus ----------------------------------------------------------------------
    M. mazei MDKKPLNTLISATGLWMSRTGTIHKIKHHEVSRSKIYIEMACGDHLVVNNSRSSRTARALRHHKYRKTCK
  M. barkeri MDKKPLDVLISATGLWMSRTGTLHKIKHHEVSRSKIYIEMACGDHLVVNNSRSCRTARAFRHHKYRKTCK
D. hafniense ----------------------------------------------------------------------

             ----------------------------------------------------------------------
             RCRVSDEDLNKFLTKANEDQTSVKVKVVSAPTRTKKAMPKSVARAPKPLENTEAAQAQPSGSKFSPAIPV
             RCRVSDEDINNFLTRSTESKNSVKVRVVSAPK-VKKAMPKSVSRAPKPLENSVSAKASTNTSRSVPSP--
             ----------------------------------------------------------------------

                                                                                 24
             ---------------------------------------------MTVKYTDAQIQRLREYGN-GTYEQK
             STQESVSVPASVSTSISSISTGATASALVKGNTNPITSMSAPVQASAPALTKSQTDRLEVLLNPKD----
             --------------------------------AKSTPNSSVPASAPAPSLTRSQLDRVEALLSPED----
             --------------------------MFLTRRDPP----------LSSFWTKVQYQRLKELNASGEQLEM

                                                                                 94
             VFEDLASRDAAFSKEMSVASTDNEKKIKGMIANPSRHGLTQLMNDIADALVAEGFIEVRTPIFISKDALA
             --EISLNSGKPFRELESELLSRRKKDLQQIYAEERENYLGKLEREITRFFVDRGFLEIKSPILIPLEYIE
             --KISLNMAKPFRELEPELVTRRKNDFQRLYTNDREDYLGKLERDITKFFVDRGFLEIKSPILIPAEYVE
             GFSDALSRDRAFQGIEHQLMSQGKRHLEQLRTVKHRPALLELEEKLAKALHQQGFVQVVTPTIITKSALA

                                                                                164
             RMTITEDKPLFKQVFWIDEKRALRPMLAPNLYSVMRDLRDHTDGPVKIFEMGSCFRKESHSGMHLEEFTM
             RMGIDNDTELSKQIFRVDKNFCLRPMLAPNLYNYLRKLDRALPDPIKIFEIGPCYRKESDGKEHLEEFTM
             RMGINNDTELSKQIFRVDKNLCLRPMLAPTLYNYLRKLDRILPGPIKIFEVGPCYRKESDGKEHLEEFTM
             KMTIGEDHPLFSQVFWLDGKKCLRPMLAPNLYTLWRELERLWDKPIRIFEIGTCYRKESQGAQHLNEFTM

                                                                                233
             LNIVDMGPRG-DATEVLKNYISVVMKAAGLPDYDLVQEESDVYKETIDVEINGQEVCSAAVGPHYLDAAH
             LNFCQMGSGCT--RENLESIITDFLNHLG-IDFKIVGDSCMVYGDTLDVMHGDLELSSAVVGPIPLDREW
             VNFCQMGSGCT--RENLEALIKEFLDYLE-IDFEIVGDSCMVYGDTLDIMHGDLELSSAVVGPVSLDREW
             LNLTELGTPLEERHQRLEDMARWVLEAAGIREFELVTESSVVYGDTVDVMKGDLELASGAMGPHFLDEKW

                                                 275
             DVHEPWSGAGFGLERLLTIREKYSTVKKGGASISYLNGAKIN--
             GIDKPWIGAGFGLERLLKVKHDFKNIKRAARSESYYNGISTNL-
             GIDKPWIGAGFGLERLLKVMHGFKNIKRASRSESYYNGISTNL-
             EIFDPWVGLGFGLERLLMIREGTQHVQSMARSLSYLDGVRLNIN
```

**Supplementary Figure 3.** Clustal Omega multiple sequence alignment of *Ma*PylRS with homologs from *M. mazei*, *M. barkeri*, and *D. hafniense*. Red boxes indicate *Ma*PylRS residues that were mutated during PANCE. The numbering corresponds to the *M. alvus* sequence.

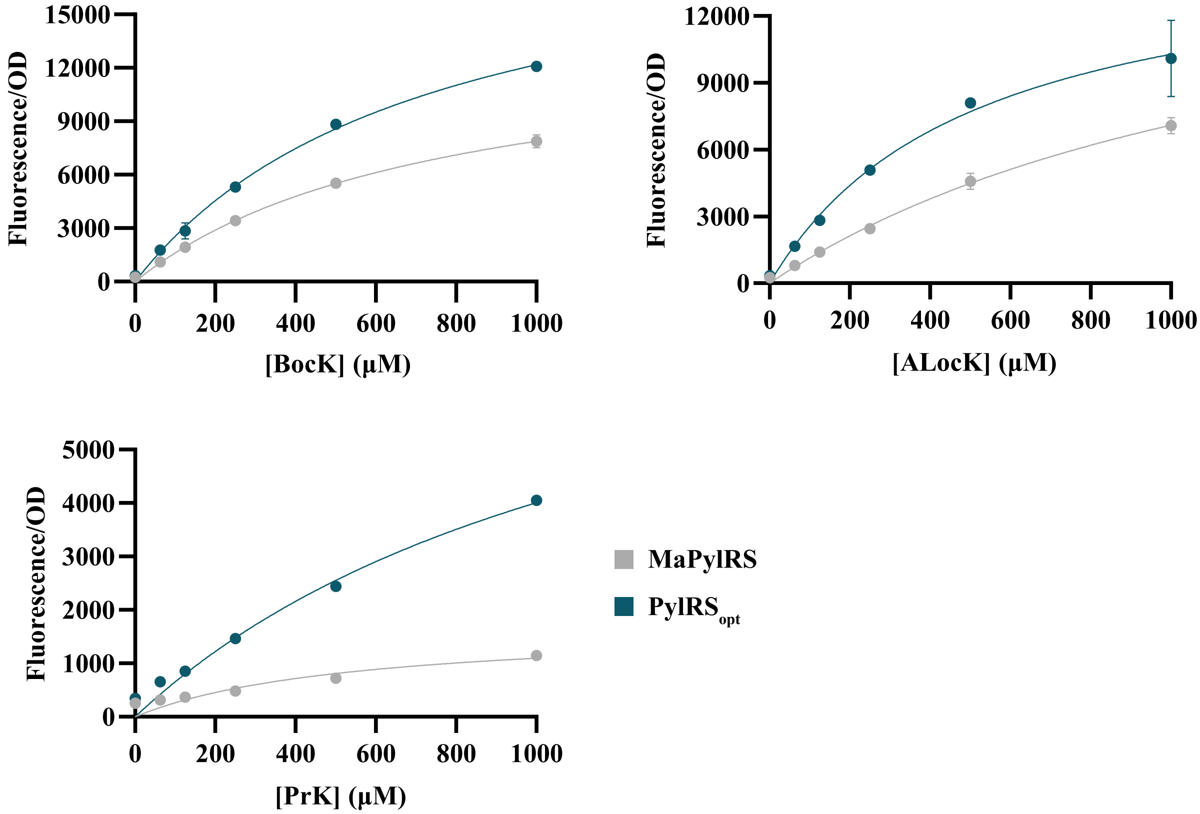


**Supplementary Figure 4.** Titration of select ncAAs into cells expressing sfGFP 2TAG, *Ma*tRNA$^{Pyl}$, and either *Ma*PylRS or PylRS$_{opt}$.
